# Evaluation of spontaneous regional brain activity in weight-recovered anorexia nervosa

**DOI:** 10.1038/s41398-020-01081-0

**Published:** 2020-11-11

**Authors:** Maria Seidel, Daniel Geisler, Viola Borchardt, Joseph A. King, Fabio Bernardoni, Charlotte Jaite, Veit Roessner, Vince Calhoun, Martin Walter, Stefan Ehrlich

**Affiliations:** 1 Faculty of Medicine, Division of Psychological and Social Medicine, and Developmental Neuroscience, Technische Universität Dresden, Dresden, Germany; 2 Clinical Affective Neuroimaging Laboratory, Magdeburg, Germany; 3 Department of Behavioral Neurology, Leibniz Institute for Neurobiology, Magdeburg, Germany; 4 Department of Child and Adolescent Psychiatry, Psychosomatics and Psychotherapy, Charité–Universitätsmedizin Berlin, Freie Universität Berlin, Humboldt-Universität zu Berlin, and Berlin Institute of Health, Berlin, Germany; 5 Faculty of Medicine, Department of Child and Adolescent Psychiatry, University Hospital C. G. Carus, Technische Universität Dresden, Dresden, Germany; 6 Tri-institutional Center for Translational Research in Neuroimaging and Data Science (TReNDS), Georgia State University, Georgia Institute of Technology, Emory University, Atlanta, GA USA; 7 Department of Psychiatry and Psychotherapy, Jena University Hospital, Jena, Germany; 8 Faculty of Medicine, Translational Developmental Neuroscience Section, Eating Disorder Research and Treatment Center, Department of Child and Adolescent Psychiatry, Technische Universität Dresden, Dresden, Germany

**Keywords:** Neuroscience, Psychology

## Abstract

Whereas research using structural magnetic resonance imaging (sMRI) reports sizable grey matter reductions in patients suffering from acute anorexia nervosa (AN) to be largely reversible already after short-term weight gain, many task-based and resting-state functional connectivity (RSFC) studies suggest persistent brain alterations even after long-term weight rehabilitation. First investigations into spontaneous regional brain activity using voxel-wise resting-state measures found widespread abnormalities in acute AN, but no studies have compared intrinsic brain activity properties in weight-recovered individuals with a history of AN (recAN) with healthy controls (HCs). SMRI and RSFC data were analysed from a sample of 130 female volunteers: 65 recAN and 65 pairwise age-matched HC. Cortical grey matter thickness was assessed using FreeSurfer software. Fractional amplitude of low-frequency fluctuations (fALFFs), mean-square successive difference (MSSD), regional homogeneity (ReHo), voxel-mirrored homotopic connectivity (VHMC), and degree centrality (DC) were calculated. SMRI and RSFC data were analysed from a sample of 130 female volunteers: 65 recAN and 65 pairwise age-matched HCs. Cortical grey matter thickness was assessed using FreeSurfer software. Fractional amplitude of low-frequency fluctuations (fALFF), mean-square successive difference (MSSD), regional homogeneity (ReHo), voxel-mirrored homotopic connectivity (VHMC), and degree centrality (DC) were calculated. Abnormal regional homogeneity found in acute AN seems to normalize in recAN, supporting assumptions of a state rather than a trait marker. Aberrant fALFF values in the cerebellum and the infertior temporal gyrus could possibly hint towards trait factors or a scar (the latter, e.g., from prolonged periods of undernutrition), warranting further longitudinal research.

## Introduction

Anorexia nervosa (AN) is a severe mental disorder characterized by an intense fear of weight gain and a distorted body image, and patients continue to engage in dietary restriction or other (compensatory) behaviours to avoid weight gain despite severe undernutrition^1,2^. Relapse rates in AN are high and long-term outcome studies generally show only low rates of full recovery^3^. Although neurobiological underpinnings of the aetiology and maintenance of the disorder are generally recognized, the exact mechanisms are still unknown^4,5^.

Neuroimaging studies on structural data show that the widespread reductions in grey matter and alterations in white matter identified in patients suffering from acute AN (acAN) reach normal levels already after short-term weight rehabilitation^6–9^. However, research looking at task-based and resting-state functional magnetic resonance imaging (fMRI) draws a more heterogeneous picture, with some studies reporting persistent functional alterations even after long-term weight recovery^10–16^. Nevertheless, mixed results regarding the exact localization and the direction of these alterations^17,18^ warrant a more comprehensive understanding of the underlying mechanisms.

Studies investigating associations between functional connectivity between different areas of the brain and spontaneous regional brain activity of the blood-oxygen-level-dependent (BOLD) signal found evidence of a close connection between the two. For instance, one study demonstrated that inter-regional resting-state functional connectivity (RSFC) was strongly linked to the amplitude of low-frequency fluctuations (ALFFs) of the BOLD signal^19^. ALFF is generally considered to reflect the magnitude of intrinsic neural activity^20,21^ and measures the intensity of spontaneous neural oscillations. Fractional ALFF (fALFF) is defined as the proportion of these low-frequency fluctuations (0.01–0.1 Hz) to the whole signal. Another more simple method to assess moment-to-moment brain signal variability is the mean-square successive difference (MSSD), based on the sum of squared difference between the BOLD signal of successive time points.

In addition to BOLD signal characteristics of single voxels, measures of local connectivity, more specifically the relational characteristics among multiple voxels, might also affect long-range connectivity and brain functioning in general^19^. For instance, regional homogeneity (ReHo) offers a measure of the regional coherence of activation in a single voxel with its neighbours^22,23^. Another measure, degree centrality (DC), reflects the strength of connection for a given voxel with all other voxels in the brain and can thus be used as an indicator of its role in transferring information across brain regions^24^. Another commonly assessed measure of brain connectivity targets correlations of low-frequency BOLD signals between bilaterally homologous brain regions. Voxel-mirrored homotopic connectivity (VMHC) calculates synchronized patterns within spatially homotopic regions of the brain^21,25^. Regions with higher VMHC have been previously interpreted as indexing increased inter-hemispheric coordinated processing^21^.

Altered spontaneous regional brain signal variability has been found in several neuropsychiatric disorders^19,26–28^. We recently found evidence of widespread alterations in both ReHo and fALFF in a large adolescent/young adult sample of acAN patients. In addition, the results pointed towards an attenuated correlation between these functional measures and cortical thickness/subcortical volume compared to healthy controls (HCs)^29^. Spontaneous regional brain activity has been linked to task-based functional activity^30,31^ or might even be used as a blueprint to explain (possibly AN related) behavioural, cognitive, and personality characteristics at an individual level^32^.

Most RSFC studies in AN, including our previous investigation of local resting measures^29^, focused on acAN and results may thus be also partially biased by consequences of insufficient intake of energy and nutrients^17^. Hence, it is still a matter of debate whether the alterations discussed above simply reflect a state marker associated with undernutrition and pseudoatrophic brain changes or whether they constitute a trait marker, possibly contributing to the aetiology of AN. Studying weight-recovered individuals with a history of AN (recAN) could therefore bring clarity to the ongoing discussion regarding state vs. trait markers in AN^33^, as differences to controls, if present, would not be attributable to acute undernutrition. Previous resting-state studies have suggested that alterations within defined networks may persist even after recovery^14,16,34^. The purpose of the current study was to test for differences in intrinsic regional brain activity between recAN and pairwise age-matched HC using voxel-wise resting-state measures including fALFF, ReHo (as reported in acAN), as well as MSSD, DC, and VHMC (as new measures of spontaneous regional brain activity). Given that previous studies have not investigated these parameters in recAN, but have established some persisting alterations in this population in both task-based neural responses and in distributed networks in classic RSFC, we hypothesized that alterations as found in our previous investigation in acAN^29^ would still be present after weight rehabilitation.

## Methods

### Participants

Data were collected from 65 recAN and a total of 70 HCs. HCs were recruited to match the samples for age. To optimize comparisons between recAN and HC, we implemented a pairwise matching algorithm^35^ in addition to the selective recruitment, resulting in a sample of 130 female volunteers: 65 recAN (15.5–29.7 years) and pairwise, age-matched 65 female HCs (15.5–29.0 years). This procedure resulted in a maximum of 0.7 years between matched pairs. To be considered ‘weight-recovered’, recAN subjects had to (1) maintain a body mass index (BMI) (kg/m^2^) > 18.5 (if older than 18 years) or above the 10th age percentile (if younger than 18 years); (2) menstruate; and (3) have not binged, purged, or engaged in restrictive eating patterns during at least 6 months before the study. On average, recAN were weight-recovered for 51 months (SD = 39), only *n* = 4 subjects fulfilled the recovery criteria for more than 6 but less than 12 months (2 recAN = 9 months, 1 recAN = 10 months, and 1 recAN = 11 months). Further details regarding the recAN sample are provided in Table 1. HC participants had to be of normal weight, eumenorrhoeic, and without any history of psychiatric illness. Exclusion criteria for both groups and possible confounding variables, e.g., the use of psychotropic medication and medical comorbidities, were obtained using the expert version of the Structured interview for anorexia and bulimia nervosa for DSM-IV (SIAB-EX), our own semi-structured research interview, and from medical records. Additional exclusion criteria for each group were a history of bulimia nervosa or ‘regular’ binge eating, psychotropic medication within 4 weeks prior to the study, substance abuse, and neurologic or medical conditions (Supplementary Material 1.1).

**Table 1:** Descriptive statistics.

Descriptive statistics						
	recAN (*N* = 65)		HC (*N* = 65)			
	Mean	SD	Mean	SD	*t*	*p*
Age	22.06	3.38	22.05	3.34	0.01	0.99
BMI	20.74	1.81	21.61	2	−2.6	0.011
BMI-SDS	−0.51	0.59	−0.22	0.58	−2.89	0.005
Duration of recovery	51.92	39.43				
EDI-2-total	171.76	46.71	136.21	26.46	5.13	<0.001
BDI-II	9.37	9.32	4.26	5.49	3.8^a^	<0.001
Leptin (ng/ml)	9.39	5.52	13.39	9.15	−2.93	0.004

*BDI-II* Beck depression inventory II, *BMI* body mass index, *BMI-SDS* BMI-SD score, *EDI-2-total* eating disorder inventory 2 total score, *HC* healthy control, *RecAN* recovered anorexia nervosa.

Results of independent samples *t*-tests, displaying mean and SD, *t* and *p*-values.

^a^Given the non-normal distribution of BDI-II data, we repeated the group comparisons using a Mann–Whitney *U*-test: *U* = 1074, *p* < 0.001). Age is given in years, duration of recovery is given in months, range: 9–168 months. Previous AN diagnoses of recovered individuals included *n* = 51 of the restrictive subtype and *n* = 14 of the binge/purge subtype.

An a priori power analysis using G*power on the basis of previously published group differences between acAN and HC^36^ gauged a sample size of *n* = 38 per group, assuming an effect size that is comparable to the group difference observed in Seidel et al.^29^ and an α-error probability of 5% (and a power of 80%).

This study was approved by the local institutional ethics review board and all participants (and their guardians if underage) gave written informed consent.

### Clinical measures

In addition to the information collected with the clinical interviews, eating disorder-specific psychopathology was assessed with the German version of the Eating Disorders Inventory (EDI-2^37^). Depressive symptoms were explored using the German version of the Beck Depression Inventory (BDI-II^38^). Participants were weighted and scaled. Assessment of the BMI was corrected for age and gender (BMI-SD score, BMI-SDS^39,40^). To quantify the degree of possible remaining undernutrition, we collected venous blood samples before the MRI scan to determine plasma leptin concentration via the commercially available software enzyme-linked immunosorbent assay (AdipoGen).

### Data acquisition

The data acquisition procedure was identical to our previous RSFC studies^14,29,41,42^. Images were acquired between 8 and 9 a.m. after an overnight fast using standard sequences with a 3T MRI scanner (TIM Trio; Siemens, Erlangen, Germany) equipped with a 12-channel head coil.

The T1-weighted structural brain scans were acquired with a rapid acquisition gradient-echo sequence: number of slices = 176; repetition time (TR) = 1900 ms; echo time (TE) = 2.26 ms; flip angle (FA) = 9°; slice thickness = 1 mm; voxel size = 1 × 1 × 1 mm^3^; field of view (FoV) = 256 × 224 mm^2^; bandwidth = 200 Hz/pixel).

Functional images were acquired by using a gradient-echo T2*-weighted echo planar imaging (EPI) with the following parameters: tilted 30° towards anterior/posterior commissure line (to reduce signal dropout in orbitofrontal regions); number of volumes = 190; number of slices = 40; TR = 2200 ms; TE = 30 ms; FA = 75°; in-plane resolution = 3.4 mm; slice thickness = 2.4 mm (1 mm gap resulting in a voxel size of 3.4 × 3.4 × 2.4 mm^3^); FoV = 220 × 220 mm^2^; bandwidth = 200 Hz/pixel. Participants were instructed to lie still with closed eyes and to stay awake during scanning.

### MRI data preprocessing

As in Seidel et al.^29^, functional and structural images were processed using the SPM8 toolbox (http://www.fil.ion.ucl.ac.uk/spm/) within the Nipype framework. We evaluated the quality of the fMRI data by manual inspection and using artefact detection tools^43^ to identify volumes with intensity outliers [>3 SDs from the mean of the time series] and excessive movement (at two thresholds: >2 mm and >1 mm in any direction). Groups did not differ in the number of outliers regarding intensity and both movement thresholds (Supplementary Table S1).

A sample-specific DARTEL template was created using structural images from all subjects^44^. The functional images were corrected for temporal slice-timing and motion simultaneously using *realign4D*^45^. The realigned files were coregistered to the subject’s structural brain image. The EPI volumes were then normalized to MNI (Montreal Neurological Institute) space using the DARTEL template and the corresponding flow field. Regression of nuisance covariates from 24-motion parameters^46^, white matter, and cerebrospinal fluid was done via the DPARSF^47^ toolbox (Version 3.2). For the current analyses, we decided to use the 24-motion parameters during nuisance regression as opposed to the 6-rigid body parameters used in our previous analyses as the standard for the main models. This decision was based on recent evidence, suggesting that this preprocessing step improves the correction of head micromovements^48^. For completeness, however, we also report the results with the previous preprocessing parameters (six-rigid body parameters) in the supplementary material (Supplementary Tables S2 and S3, method B). Additional analyses validating the initial results by using different preprocessing pipelines (see Supplementary Table S2) included a method applying nuisance regression of white matter and cerebrospinal fluid signal via the CompCor^49^ method (method C) and adding global mean signal regression (GSR, method D). Values for the MSSD, DC, and VHMC were further filtered for low frequencies (0.01–0.1 Hz). The groups showed similar global signal values [recANmean = 859.35), HCmean = 867.6), two-sample *t*-test: *t*(128) = −0.27, *p* > 0.05).

Preprocessing of T1-weighted structural brain scans and estimation of cortical grey matter thickness and subcortical grey matter volumes was carried out using FreeSurfer software (http://surfer.nmr.mgh.harvard.edu, version 5.1.0). For more detail refer to Bernardoni et al.^50^ or the Supplementary Material 1.2.

### ALFF/fALFF, MSSD, ReHo, DG, and VHMC calculation

ALFF/fALFF, MSSD, ReHo, DC, and VHMC values were calculated using DPARSF Version 3.2^47^. ALFF is the averaged square root of the amplitude of the BOLD time series within a specific low-frequency range (0.01–0.1 Hz). fALFF is defined as the division of ALFF within the specified frequency band (0.01–0.1 Hz) by the entire frequency range observed in the signal^20^. Following our previous analytic approach in acAN^29^, we report fALFF values, which are considered to be less susceptible to physiological noise^20^. In a supplementary analysis, fALFF values were calculated within the more narrowly defined frequency bands usually reported, Slow-4 (0.27–0.073 Hz) and Slow-5 (0.01–0.27 Hz)^21^.

For the calculation of MSSD, preprocessed resting-state fMRI data were smoothed (Gaussian kernel of 6 mm at full width half maximum) and normalized to z-statistics by subtracting the standard deviation of each voxel’s time series from its mean^26,27^. MSSD was then calculated by squaring the difference from timepoint *t* to timepoint *t* + 1. The squared values across the entire time series were then averaged to produce a single MSSD metric for each voxel of each subject^51^.

ReHo estimation was done on a voxel-by-voxel basis by calculating Kendall’s coefficient of concordance, which estimates similarity in the time series of a given voxel to its nearest 26 voxels based on the ReHo hypothesis^22^.

DC was calculated as the Pearson’s correlation coefficients between the time series of each grey matter voxel with all others, which results in an individual whole-brain functional connectivity map. As in Buckner et al.^24^, we restricted analysis to positive correlations above a threshold of *r* = 0.25. This threshold was chosen to eliminate voxels with weak correlations that can be associated to signal noise or white matter^24^. Subsequently, DC was computed for each voxel as the number of significant correlations (binarized DC) or as the sum of the weights of the significant connections (weighted DC). Pearson’s correlations were also computed between the time series of every pair of mirrored inter-hemispheric voxels to calculate VHMC. The resulting correlations for each paired voxel produced a VMHC whole-brain map.

Prior to second-level analyses, subject-level voxel-wise fALFF, ReHo, DG, and VMHC maps were standardized into subject-level z-score maps. With the exception of the data used for the calculation of the MSSD, smoothing was applied after calculation of each parameter with a Gaussian kernel of 6 mm at full width half maximum. A grey matter mask (obtained from the MNI template with a threshold of a probability higher than 0.3) was used to remove non-brain tissue in all maps.

### Statistical analysis

Differences between recAN and HC in fALFF, MSSD, ReHo, DC, and VHMC values were obtained using independent (voxel-wise) two-sample *t*-tests in SPM8. We corrected for multiple comparisons by applying family-wise error rate (*p* < 0.05) at cluster level, with a cluster forming threshold of *p* < 0.001^52^. All initial analyses were supplemented by a model with age as covariate, as well as using different preprocessing methods for validation of the results as stated above. For details on an additional analysis also including data of acAN from our previous publication^29^, please refer to Supplementary Material 1.6.

Group differences in questionnaire data (EDI-2 and BDI-II), BMI-SDS, and plasma leptin were determined via independent samples *t*-test or Mann–Whitney *U*-test, if data were not normally distributed (BDI-II), using SPSS 23 software. If variance was not equal between groups, corrected *p*-values are reported. Next, we explored the associations between measures of intrinsic brain activity in regions of interest (ROIs) and clinical symptoms (EDI-2 and BDI-II), BMI-SDS, and plasma leptin. ROIs were built on the basis of clusters in which we detected significant group differences in fALFF values. The relationship between the ROIs and clinical variables were assessed using Pearson’s correlations, (or Spearman’s *ρ* for correlations with BDI-II, as data were not normally distributed), for each group separately. To this end, parameter estimates (betas) were extracted and averaged from ROIs with MarsBaR^53^. We defined ROIs by thresholding the two-sample *t*-tests of group differences with an uncorrected voxel-wise threshold of *p* < 0.001.

To investigate whether the findings of different structure–function relationships in acAN were also evident in recAN, we explored correlations between cortical grey matter thickness and subcortical grey matter volumes and fALFF or ReHo values. Pearson’s correlations were calculated using extracted fALFF and ReHo values of all 34 anatomical cortical labels of the Desikan–Kiliany atlas^54^ and 7 subcortical regions (Supplementary Material 1.3).

## Results

### Demographics and clinical variables

As displayed in Table 1, recAN participants did not differ from their HC counterparts in age, but still had lower BMI-SDS, plasma leptin values, and some residual eating disorder (EDI-2) and depression symptoms (BDI-II).

### Group differences in fALFF, ReHo, DC, VHMC, and MSSD

**Fig. 1:**
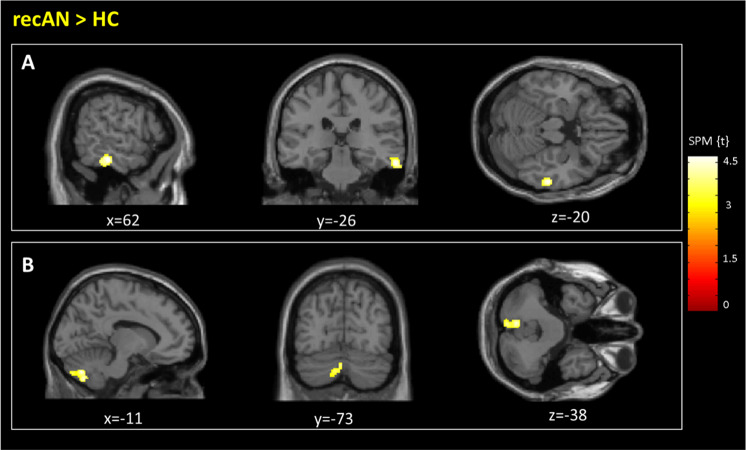
Brain regions showing differences in fALFF values for recAN participants compared to HC (FWE corrected, *p* < 0.05). **a** Inferior temporal gyrus [62–28–20], *t* = 4.72. **b** Cerebellum [−8–70–36], *t* = 4.66. fALFF, fractional amplitude of low-frequency fluctuations; HC, healthy control; recAN, recovered anorexia nervosa. Differences in fALFF Values between weight-recovered and healthy individuals.

RecAN showed significantly elevated fALFF values in one cluster in the right inferior temporal gyrus and one cluster in the left cerebellum (Fig. 1). fALFF results of further analyses with alternative preprocessing methods (B: six-rigid body parameters (the method used in the acAN sample in previous research), C: CompCor^49^ method, D: with GSR; see also ‘Methods’) were highly similar (Supplementary Table S2). The same was the case when a narrower frequency band for fALFF was considered (Slow-4; however, there were no group differences for Slow-5; see Supplementary Table S2: method E and F) or when age was added as a covariate to the model (Supplementary Table S2: method G).

DC values did not differ between groups applying the standard preprocessing procedure used in the current study (Supplementary Table S3, method A). However, DC values where higher in recAN than HC in a cluster in the somatosensory cortex (Supplementary Fig. S1) using the preprocessing method (B) as in our previous paper on acAN (Supplementary Table S3). ReHo, VHMC, and MSSD did not differ between the two groups regardless of preprocessing method.

Post-hoc supplementary analyses using Bayesian independent samples t-test were applied to further investigate the amount of evidence for the absence of group differences between recAN and HC within clusters previously found to be significantly different between acAN and HC (acAN > HC and acAN < HC) for fALFF and ReHo values (Supplementary Material 1.5). Bayesian results did not show reliable evidence that either the null hypothesis or the alternative hypothesis was true for fALFF values in regions identified by the acAN < HC contrast (BF_01_ = 0.98; BF_10_ = 1.02). However, for brain regions defined using the acAN > HC contrast of our previous paper, we found strong evidence for the alternative hypothesis (BF_10_ = 11.95), with recAN showing increased fALFF values compared to HC as reported above. Results showed moderate evidence in favour of the the null hypothesis (BF_01_ = 3.43, BF_01_ = 5.21), indicating no difference between recAN and HC for ReHo values (Supplementary Table S4). When comparing fALFF and ReHo values between all three groups (acAN, recAN, and HC) in one analysis, the results remained the same (Supplementary Table S5).

### Associations with clinical variables and grey matter structure

No significant correlations between the extracted fALFF betas in either of the identified clusters in the cerebellum or inferior temporal gyrus and clinical parameters such as duration of recovery, BMI-SDS, leptin, EDI-2, or BDI-II were evident (Supplementary Table S6).

Associations between structural measures (cortical thickness/subcortical volumes (34 cortical and eight subcortical ROIs) and fALFF or ReHo values did not show any clear pattern within the groups, hemispheres, or parameters (Supplementary Table S7). Following the approach taken in our previous study investigating acAN patients^29^, we averaged absolute values of correlation coefficients across participants for each ROI within each hemisphere for each group (for more details, see Supplementary Material 1.2). Averaged absolute correlation coefficients did not differ between recAN and HC (fALFF: F(1,82) = 1.95, *p* > 0.05; ReHo: F(1,82) = 1.12, *p* > 0.05). Further, cortical thickness was not different between groups in the ROIs that overlapped with clusters in which we reported differences between recAN and HC in fALFF values or any other ROI from the Desikan–Kiliany atlas (Table S8).

## Discussion

The aim of the present study was to investigate different characteristics of sponteaneous regional brain activity in weight-recovered individuals with a history of AN (recAN). Previous analyses of intrinsic brain activity including ReHo and fALFF in acutely underweight AN (acAN) patients using the same general study and analysis design showed widespread alterations compared to HCs^29^. The relative absence of differences between the recAN and HC samples in most of the parameters investigated here, point towards a partial normalization of those alterations after weight recovery. Only fALFF values indicated some alterations in recAN in regions of the inferior temporal gyrus and the cerebellum. In line with these findings, post-hoc Bayesian analyses showed evidence in favour of the null hypothesis suggestive of relative normalization in recAN for ReHo values, but not for fALFF in the brain regions in which we previously found acAN and HC to differ. However, using a second measure of signal variability (MSSD), which we had not included in our previous work, no alterations were evident in recAN. Similarly, the reduced structure–function relationship between cortical thickness/subcortical volume and fALFF and ReHo measures that we previously found in acAN was also not detected in the current recAN-HC comparison.

The observed group difference in fALFF located in the inferior temporal gyrus showed close proximity to a group difference we previously observed in acAN in ReHo values^29^. The inferior temporal gyrus as part of the ventral visual stream is involved in higher-order object processing^55^. Multiple fMRI studies have revelaed alterations in this pathway both at rest and during visual presentation of face, food or body stimuli in adult AN and individuals with body dysmorphic disorder^34,56–62^. Interestingly, the infertior temporal gyrus also shows similar alteration in fALFF values in individuals with autism spectrum disorder^63^, who share deficits in social cognition^64,65^. Further research targeting this particular area is warranted to investigate whether intrinsic brain characteristics may constitute a potential trait variable or ‘scarring’ factor in the disorder.

Group differences in fALFF were also evident in the cerebellum, more specifically in the vermis, which has also been associated with feeding behaviour^66^. AN patients have been found to show increased activation in this part of the cerebellum after an overnight fast^11^, as well as decreased activation while viewing food pictures in the sated state^67^, highlighting it’s role in food processing. Some research has also indicated that grey matter volume loss in the cerebellum of AN patients might persist even after recovery^7,68^. Moreover, cerebellum grey matter has been associated with clinical outcome^68^ (in adolescents) and illness duration^69,70^. Therefore, it has been suggested that volume changes in the cerebellum may play an important, potentially underestimated, role in AN^71^. Although we did not detect any structural alterations in either of the identified regions of the cerebellum or the inferior temporal gyrus nor any significant relationship with fALFF, we cannot completely rule out the possibility of persistent (micro-)structural changes, which might explain altered fALFF values in these regions in former AN patients.

Additional voxel-based measures, such as DC, reflecting associations between BOLD activity in single voxels and all other voxels in the brain appeared to be higher in recAN participants in the somatosensory and premotor regions, but only when certain preprocessing methods were applied. One previous investigation of DC reported group differences in the inferior frontal gyrus between acAN patients and HC^72^, using preprocessing methods (including the six-rigid body parameters during nuisance regression) similar to those with which group differences were evident in our acAN dataset^29^. Additional measures looking at inter-hemispheric synchronicity (VHMC) did not show any differences between groups. Overall, the group differences observed in the current study were rather subtle. These findings obtained with measures of brain function mostly mirror those we have previously found using measures of brain structure^7–9,50^; underlining the impressive capacity of the brain to recover even after prolonged periods of undernutrition.

The current results have to be considered in light of several important limitations. Given the systematic age differences between acutely ill patients from our previous manuscript and the recovered sample of this study, comparing all three groups was not part of our primary statistical analysis. Our study was intentionally designed in this manner with the purpose of conducting separate (but nonetheless identical) analyses adressing effects of maturation as well as state vs. trait effects. Due to the the cross-sectional study design, it is difficult to draw definitive conclusions regarding the question whether the remaining group differences represent trait effects or ‘scarring’ from the acute underweight state of the disorder. A longitudinal research design might shed more light on whether alterations are a result or a potential precursor of pathological behaviour. Further limitations include the general reliability and validity of resting-state measures that have been a matter of debate, in particular their susceptibility to different acquisition and preprocessing methods^73–76^. Similarly, processing the structural data in a different way, e.g. using voxel-based morphometry might affect the potential to detect associations with resting-state measures.

Taken together, the group differences in basic BOLD signal characteristics of single voxels as well as relational characteristics among multiple voxels previously detected between acAN and HC^29^ seem to largely disappear during recovery. Although we provide evidence for a relative normalization of some of these measures, for others, e.g., fALFF, the extent of normalization remains an open question. Overall, the presented evidence suggestive of normalization of spontaneous regional brain activity and connectivity may send a positive message to patients and could be useful information for patient education and psychotherapy^77^.

## Supplementary information

Supplementary Material
