# Supplementary material for: Evaluation of spontaneous regional brain activity in weight-recovered anorexia nervosa
Source: Transl Psychiatry. 2020 Nov 11;10:395. doi: 10.1038/s41398-020-01081-0 (PMC7658198; doi:10.1038/s41398-020-01081-0)
Supplement: Supplementary file 1 — Supplementary Material [file 41398_2020_1081_MOESM1_ESM.docx]

Seidel et al. Evaluation of spontaneous regional brain activity in weight-recovered anorexia nervosa.

**Supplementary Material**

- 1. Participant exclusion criteria

Participants of both groups (recAN and HC) at the focus of the current analyses were excluded if they had a history of organic brain syndrome, schizophrenia, substance dependence, psychosis NOS, bipolar disorder, bulimia nervosa or binge-eating disorder. Further exclusion criteria included IQ<85, psychotropic medication within 6 weeks prior to the study, current substance abuse, inflammatory, neurologic or metabolic illness, chronic medical or neurological illness that could affect appetite, eating behavior, or body weight, clinically relevant anemia, pregnancy or breast feeding. Inclusion criteria for HC included normal lifetime BMI (kg/m2)>18.5 (if older than 18 years) or above the 10th age percentile (if younger than 18 years). 31 (47.7%) of recAN and 22 (33.8%) of HC participant data were also included in our previous publication of resting state data in recAN ^1^. Out of 65 individuals with a history of AN, only four had maintained their BMI for less than 12 months (9 months=2, 10 months=1, 11 months=1). The majority of recovered participants had previously been patients at the child and adolescent psychiatry department of the collaborating clinic and had also taken part as acute patients in previous parts of the study. Further, participants with a history of AN were recruited via advertising through email lists at the university, AN related homepages and public paper handouts.

- 1. Preprocessing of structural MRI data using FreeSurfer

Surface reconstruction was first performed for each hemisphere separately, including tessellation of the gray matter-white matter boundary, automated topology correction, and surface deformation following intensity gradients to optimally place the gray-white and gray-cerebrospinal fluid borders at the location where the greatest shift in intensity defines the transition to the other tissue class. The quality of the surface reconstruction and segmentation was assured by visual inspection by a trained examiner with the support of quality assurance tools implemented in FreeSurfer (https://surfer.nmr.mgh.harvard.edu/fswiki/QATools) and exploratory analysis of the parcellation and segmentation statistics for outliers as in our previous studies ^2,3^. If quality could not be assured the participant was not included in the analyses. The resulting surfaces were then used to calculate CT at each vertex as the closest distance from the gray-white boundary to the pial surface. For the subcortical volumetric analyses, we used the automated segmentation procedures implemented in the FreeSurfer pipeline ^4^ to assign an anatomical label to each voxel based on probabilistic information estimated from a manually labeled training set.

- 1. Calculation of structure function relationship

To calculate correlations between fALFF/ReHo values and cortical thickness, we extracted averaged cortical thickness measurements from each of the 34 regions labelled by the Desikan-Killiany cortical atlas ^5^ and eight subcortical grey matter volumes (amygdala, insula, palladium, caudate, hippocampus, brainstem, putamen, cerebellum) for each subject in each hemisphere [42 x 2 regions of interest (ROIs)].

To compare the structure-function relationship between the groups, we averaged the absolute value of correlation coefficients across participants for each ROI within each hemisphere for each group. We then calculated a 2×2 repeated measures ANOVA with hemisphere as within-subject and group as between-subject factor to compare averaged correlation coefficients across all ROIs. Single measurements were the averaged coefficients for each group in the single ROIs.

- 1. Calculation of association between clinical characteristics and resting state parameters

We explored the association between clusters which showed significant group differences and clinical symptoms (EDI-2, BDI-II), BMI-SDS as well as plasma leptin. The relationship was assessed using Pearson’s correlations for each group separately using SPSS 23 software. In order to do so, parameter estimates (betas) were extracted and averaged from ROIs with MarsBaR ^6^. We defined ROIs by thresholding the two-sample t-tests of group differences with an uncorrected voxel-wise threshold of p<0.001.

- 1. Calculation of Bayesian independent t-test

In order to investigate processes of normalization between recAN and HC in fALFF and ReHo values we tested the evidence to support the null hypothesis (no group difference). In order to do so we calculated Bayesian independent samples t-test ^7^ on extracted beta values of brain regions that were previously established to show significant differences between acutely ill AN patients (acAN) and HC ^8^. T-tests were calculated on averaged clusters for each parameter and contrast (either acAN>HC or acAN>HC). For results see Table S4.

- 1. Exploratory comparisons between acAN, recAN, HC

In order to compare fALFF and ReHo values between acAN, recAN and HC in one single statistical analysis, we used brain masks based on significant group differences in fALFF and Reho values between acAN and HC as identified in the previous publication ^8^. To extract beta estimates within these masks from all groups acAN, recAN and HC (acANn=74, recANn=65, HCn=122) Marsbar ^6^ was used. To compare the values between the groups we calculated an ANCOVA with age as covariate. For results see Table S5.

**Supplementary Figures**

**Figures S1**

**
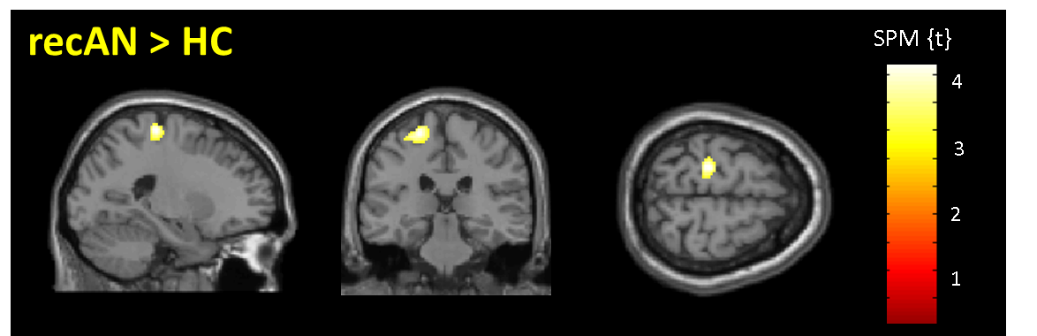
**

Figure S1: Brain regions showing differences in DC values for recAN participants compared to HC using preprocessing method B (see Table S3) in the somatosensory cortex [-22 -28 66]. Values are FWE corrected, p<0.05. DC=degree centrality (positive binarized values), HC=healthy control; recAN=recovered anorexia nervosa.

**Supplementary Tables**

**Table S1:** **Results of Independent Sample T-tests for Group Comparisons of Number of Intensity and Motion Outliers**

| **Outlier** |  |  |  |  |  |  |
| --- | --- | --- | --- | --- | --- | --- |
| **Threshold** |  | **recAN** | **HC** |  | **t** | **p** |
| 2 mm |  | 0.11 | 0.43 |  | -1.58 | 0.12 |
| 1 mm |  | 0.57 | 1.57 |  | -1.43 | 0.16 |

Table S1: Results of independent sample t-tests for group comparisons of number of intensity and motion outliers with a threshold of 2 mm and 1 mm for motion outliers. Volumes that exceeded an intensity threshold of three standard deviations or a threshold of 2 mm/1 mm normalized movement in any direction were classified as outliers. recAN=recovered anorexia nervosa, HC=healthy control.

**Table S2: Brain Regions Showing Differences in Amplitude of Low Frequency Fluctuation Values for Recovered Anorexia Nervosa Patients Compared to Healthy Controls with Different Preprocessing Methods and Covariates**

| **fALFF** |  |  |  |  |  |  |  |
| --- | --- | --- | --- | --- | --- | --- | --- |
|  | Label | p (cluster) | k | t(peak) | x | y | z |
|  | *recAN > HC* | |  |  |  |  |  |
| **A) 24-motion parameters** | Inferior Temporal Gyrus | 0.02 | 148 | 4.72 | 62 | -28 | -20 |
|  | Cerebellum | <0.001 | 235 | 4.66 | -8 | -70 | -36 |
| **B) 6-rigid body parameters** | Inferior Temporal Gyrus | 0.04 | 121 | 4.44 | 62 | -28 | -22 |
|  | Cerebellum | <0.001 | 318 | 4.80 | -10 | -68 | -44 |
| **C) With CompCor, 24-motion parameters** | Inferior Temporal Gyrus | 0.01 | 162 | 4.98 | 62 | -26 | -22 |
|  | Cerebellum | <0.001 | 270 | 4.45 | -10 | -68 | -44 |
| **D) With GSR, 24-motion parameters** | Inferior Temporal Gyrus | 0.03 | 137 | 4.64 | 62 | -28 | -20 |
|  | Cerebellum | <0.001 | 324 | 4.74 | -10 | -68 | -44 |
| **E)** **Slow-4, 24-motion parameters** | Inferior Temporal Gyrus | 0.04 | 120 | 4.61 | 62 | -28 | -20 |
|  | Cerebellum | <0.001 | 192 | 4.50 | -8 | -70 | -36 |
| **F) Slow-5, 24-motion parameters** | Inferior Temporal Gyrus | N.S. |  |  |  |  |  |
|  | Cerebellum | N.S. |  |  |  |  |  |
| **G) Age as covariate, 24-motion parameters** | Inferior Temporal Gyrus | 0.02 | 147 | 4.72 | 62 | -28 | -20 |
|  | Cerebellum | <0.001 | 241 | 4.65 | -8 | -70 | -36 |

Table S2: fALFF values are calculated with alternative methods of preprocessing: A) Standard preprocessing as described in detail in the main manuscript, using 24-motion parameters during nuisance regression, B) using 6-rigid-body parameters during nuisance regression as used in our previous report on acute AN patients ^8^, C) Using the CompCor Method during nuisance regression, D) adding GSR during nuisance regression. Method E ) represents fALFF values based on the more narrow frequency band Slow-4 calculated with method A preprocessing. F) represents fALFF values based on the more narrow frequency band Slow-5 calculated with method A preprocessing. G) represents the method presented in the main manuscript but with age as covariate. Clusters were family-wise error (FWE, *p*<.05) corrected for multiple testing. *t*-tests were one-tailed. Coordinates in Montreal Neurological Institute (MNI) space [x,y,z]. fALFF=fractional amplitude of low frequency fluctuations; GLM=general linear model, GSR=global signal regression, HC=healthy control, recAN=recovered anorexia nervosa.

**Table S3: Degree Centrality with GSR during Preprocessing**

| **Degree Centrality** |  |  |  |  | |  | | |  |  |
| --- | --- | --- | --- | --- | --- | --- | --- | --- | --- | --- |
|  | Label | p(cluster) | k | t (peak) | x | | y | z | | |
|  | *recAN >HC* |  |  |  |  | |  |  | | |
| **A) 24-motion parameters, low-pass filter, no GSR** | Somatosensory cortex | N.S. |  |  |  | |  |  | | |
| **B) 6-rigid-body parameters, no low-pass filter, no GSR** | Somatosensory cortex | 0.03 | 215 | 4.44 | -22 | | -28 | 66 | | |
| **C) With CompCor, Friston 24, low-pass filter, no GSR** | Somatosensory cortex | N.S. |  |  |  | |  |  | | |
| **D) With GSR, Friston 24, low-pass filter** | Somatosensory cortex | N.S. |  |  |  | |  |  | | |
| **E) Age as covariate, 24-motion parameters, low-pass filter** | Somatosensory cortex | N.S. |  |  |  | |  |  | | |

Table S3: DC values were calculated with alternative preprocessing methods. A) Standard preprocessing as described in detail in the main manuscript, using 24-motion parameters during nuisance regression, as well as low-pass filtering (0.01-0.1Hz), B) Using 6-rigid-body parameters during nuisance regression and no low-pass filtering as used in our previous report on acute AN patients ^8^, C) Using the CompCor Method during nuisance regression, D) adding GSR during nuisance regression. Method E) represents the method presented in the main manuscript but with age as covariate, Clusters were family-wise error (FWE, *p*<.05) corrected for multiple testing. *t*-tests were one-tailed. None of the clusters would not have survived the application of a two-tailed threshold. Coordinates in Montreal Neurological Institute (MNI) space [x,y,z]. DC=degree centrality (binarized values, positive weighted values show the same pattern); GSR=global signal regression; HC=healthy control; recAN=recovered anorexia nervosa.

**Table S4: Results of Bayesian Independent Sample T-tests for Group Comparisons of averaged fALFF and ReHo values**

| **Bayesian Independent Samples T-Test** | | | |
| --- | --- | --- | --- |
| **Cluster** | **BF₀₁** | **BF₁₀** | **Error %** |
| **fALFF_acAN>HC_** | 0.084 | 11.951 | 2.629e -7 |
| **fALFF_acAN<HC_** | 0.976 | 1.024 | 7.920e -5 |
| **ReHo_acAN>HC_** | 3.426 | 0.292 | 0.036 |
| **ReHo_acAN<HC_** | 5.206 | 0.192 | 2.582e -6 |

Table S4: Averages were estimated on basis of extracted beta values from clusters with significant group differences in our previous study on acute patients ^8^. fALFF_acAN>HC_ consists of ROIs Hippocampus/Parahippocampus right hemisphere, Hippocampus/Parahippocampus left hemisphere, Occipital Pole/Lingual Gyrus left hemisphere, Midbrain in which we had found group differences in fALLF in the contrast acAN>HC. fALFF _acAN<HC_ consists of Middle Frontal Gyrus left hemisphere, Middle Frontal Gyrus right hemisphere in which we had found acAN<HC. ReHo_acAN>HC_ consists of ROIs Thalamus/Caudate bilateral, Superior Temporal Gyrus/Insula right hemisphere, Occipital Gyrus/Lingual Gyrus bilateral, Hippocampus/Pons right hemisphere, Brainstem in which we had found acAN>HC. Reho_acAN<HC_ consists of ROIs Inferior Temporal Gyrus/Fusiform Gyurs left hemisphere, Inferior Temporal Gyrus right hemisphere, Inferior Temporal Gyrus/Fusiform Gyurs left hemisphere, Cerebellum Posterior Lobe left hemisphere, and Cerebellum Posterior/Anterior Lobe left hemisphere in which we had found acAN<HC. For exact location and size of clusters please refer to Seidel et al. ^8^. BF_01_ indicates the strength of evidence for the null hypothesis namely that there is no group difference. BF_10_ indicates the strength of evidence for the alternative hypothesis, namely that there is a group difference^7^. BF=bayes factor, ROI=region of interest, acAN=acute anorexia nervosa, HC=healthy control.

| **Cluster** | **acAN_mean_**  **(SE)** | **recAN_mean_**  **(SE)** | **HC_mean_**  **(SE)** | **F** | **Pairwise comparisons (Bonferroni corrected)** | | |
| --- | --- | --- | --- | --- | --- | --- | --- |
| **fALFF** |  |  |  |  |  |  |  |
| Hippocampus/ parahippocampal Gyrus Left | -0.03 (0.01) | -0.07  (0.02) | -0.13 (0.01) | 18.64 | acAN > HC | recAN > HC | n.s. |
| Occipital Pole/Lingual gyrus left | 0.27  (0.02) | 0.21  (0.03) | 0.11  (0.02) | 16.59 | acAN > HC | recAN > HC | n.s. |
| Midbrain | 0.27  (0.03) | 0.20  (0.03) | 0.13  (0.02) | 8.83 | acAN > HC | n.s. | n.s. |
| Hippocampus/parahippocampal Gyrus Right | -0.09 (0.01) | -0.16  (0.01) | -0.21 (0.01) | 27.15 | acAN > HC | recAN > HC | acAN > recAN |
| Middle Frontal Gyrus Left | -0.28 (0.02) | -0.16  (0.03) | -0.09  (0.02) | 20.17 | acAN < HC | n.s. | acAN < recAN |
| Middle Frontal Gyrus Right | -0.27 (0.02) | -0.14  (0.02) | -0.12 (0.01) | 20.82 | acAN < HC | n.s. | acAN < recAN |
| **ReHo** |  |  |  |  |  |  |  |
| Thalamus/Caudate Left/Right | 0.26  (0.03) | 0.01  (0.03) | 0.00 (0.02) | 24.53 | acAN > HC | n.s. | acAN > recAN |
| Occipital Gyrus/Lingual Gyrus Left/Right | 0.58  (0.04) | 0.34  (0.04) | 0.25 (0.03) | 22.53 | acAN > HC | n.s. | acAN > recAN |
| Hippocampus/Pons Right | -0.01 (0.02) | -0.17  (0.02) | -0.22 (0.01) | 39.51 | acAN > HC | n.s. | acAN > recAN |
| Brainstem | 0.07  (0.05) | -0.34  (0.06) | -0.29 (0.04) | 17.21 | acAN > HC | n.s. | acAN > recAN |
| Superior Temporal Gyrus/Insula Right | 0.08  (0.03) | -0.07  (0.03) | -0.11 (0.02) | 20.88 | acAN > HC | n.s. | acAN > recAN |
| Inferior Temporal Gyrus | -0.53  (0.022) | -0.36 (0.023) | -0.36 (0.016) | 20.65 | acAN < HC | n.s. | acAN < recAN |
| Inferior Temporal Gyrus/Fusiform Gyrus Left | -0.56 (0.02) | -0.41  (0.02) | -0.39 (0.02) | 19.17 | acAN < HC | n.s. | acAN < recAN |
| Inferior Temporal gyrus | -0.53 (0.03) | -0.36  (0.03) | -0.36 (0.02) | 16.52 | acAN < HC | n.s. | acAN < recAN |
| Cerebellum Posterior Lobe Left | -0.02 (0.03) | 0.14  (0.04) | 0.2 (0.02) | 15.2 | acAN < HC | n.s. | acAN < recAN |
| Cerebellum Posterior Lobe/Anterior Lobe Left | -0.09 (0.03) | 0.1  (0.03) | 0.12 (0.02) | 15.43 | acAN < HC | n.s. | acAN < recAN |

**Table S5: Results of univariate analyses of covariance comparing fALFF and ReHo values between acutely ill patients, remitted individuals and healthy controls**

Table S5: Results of separate univariate ANCOVAS comparing extracted betas within regions of interest between acute anorexia nervosa patients, remitted anorexia individuals and healthy controls and age as covariate. Analyses included acANn=74, recANn=65, HCn=122. All F-tests were significant. Pairwise comparisons were Bonferroni corrected for multiple testing. fALFF and ReHo were extracted from clusters previously identified to be statistically significant between acute anorexia nervosa patients and healthy controls (published in Seidel et al, 2019). fALFF=fractional amplitude of low frequency fluctuations, ReHo=regional homogeneity, acAN=acute anorexia nervosa, recAN=remitted anorexia nervosa, HC=healthy control, SE=standard error.

**Table S6: Associations between Extracted fALFF Values and Clinical Variables**

|  | **BMI-SDS** | **EDI-2-total** | **BDI-II** | **Duration of**  **Recovery** | **Leptin** |
| --- | --- | --- | --- | --- | --- |
| ***recAN*** |  |  |  |  |  |
| **Cerebellum** | -0.10 | -0.21 | -0.1 | -0.03 | -0.05 |
| **Inferior Temporal Gyrus** | -0.06 | 0.11 | 0.14 | 0.14 | 0.04 |
| ***HC*** |  |  |  |  |  |
| **Cerebellum** | 0.04 | 0.22 | 0.28 | - | 0.04 |
| **Inferior Temporal Gyrus** | 0.16 | -0.06 | 0.01 | - | 0.02 |

Table S6: Pearson correlation coefficients (and Spearman’s rho for all correlations with BDI given it’s non-normal distribution) between fALFF values extracted from clusters with significant group differences and clinical variables as measured using EDI-2-total score for AN symptoms, BDI-II for depressive symptoms, BMI-SDS, as well as plasma leptin for both groups. recAN=recovered anorexia nervosa, EDI-2=eating disorder inventory, BDI-II=Beck depression inventory, BMI-SDS=body-mass index standard deviation score, fALFF=fractional amplitude of low frequency fluctuations, none of the clusters were significant after Bonferroni correction for multiple comparisons (uncorrected p=0.03-0.91).

**Table S7: Associations between Cortical Thickness/Subcortical Volume and fALFF and ReHo Values**

|  | **fALFF** |  |  |  | **Reho** |  |  |  |
| --- | --- | --- | --- | --- | --- | --- | --- | --- |
| **ROI** | **left hemisphere**  **recAN** | **left hemisphere**  **HC** | **right hemisphere**  **recAN** | **right hemisphere HC** | **left hemisphere recAN** | **left hemisphere HC** | **right hemisphere recAN** | **right hemisphere HC** |
| **Caudalanteriorcingulate** | -0.05 | -0.15 | 0.03 | -0.12 | 0.08 | -0.15 | -0.08 | 0.00 |
| **Caudalmiddlefrontal** | -0.1 | -0.05 | -0.37** | -0.14 | -0.00 | 0.23 | -0.17 | -0.08 |
| **Entorhinal** | 0.12 | 0.02 | 0.18 | 0.01 | 0.26 | -0.16 | 0.06 | -0.03 |
| **Frontalpole** | 0.15 | 0.32* | -0.12 | 0.1 | -0.08 | 0.13 | -0.14 | 0.12 |
| **Fusiform** | 0.17 | 0.13 | 0.05 | -0.07 | 0.19 | 0.08 | 0.00 | -0.12 |
| **Inferiorparietal** | 0.07 | 0.04 | 0.03 | 0.15 | 0.01 | -0.08 | -0.22 | 0.06 |
| **Inferiortemporal** | 0.21 | -0.1 | 0.17 | 0.01 | 0.26 | 0.04 | 0.13 | -0.04 |
| **Insula** | -0.29* | 0.04 | -0.6 | 0.06 | 0.02 | -0.00 | -0.05 | -0.08 |
| **Lateraloccipital** | 0.07 | 0.23 | 0.13 | 0.04 | 0.07 | 0.17 | 0.1 | 0.03 |
| **Lateralorbitofrontal** | -0.19 | 0.05 | -0.01 | -0.05 | -0.03 | 0.12 | -0.04 | 0.01 |
| **Lingual** | -0.3 | 0.12 | 0.13 | 0.2 | 0.02 | -0.1 | 0.11 | 0.02 |
| **Medialorbitofrontal** | 0.1 | 0.2 | 0.08 | 0.16 | 0.04 | 0.06 | -0.2 | 0.19 |
| **Middletemporal** | 0.29* | 0.02 | 0.02 | 0.07 | 0.11 | 0.12 | 0.01 | 0.22 |
| **Parahippocampal** | -0.09 | 0.01 | -0.3 | 0.00 | -0.24 | -0.15 | -0.03 | 0.02 |
| **Paracentral** | -0.2 | -0.3* | 0.01 | -0.18 | -0.17 | -0.26* | -0.02 | 0.05 |
| **Parsopercularis** | -0.05 | -0.11 | 0.14 | 0.00 | 0.07 | -0.15 | 0.06 | 0.09 |
| **Parsorbitalis** | 0.1 | 0.11 | 0.15 | 0.03 | 0.26 | 0.27* | 0.1 | 0.14 |
| **Parstringularis** | 0.14 | -0.07 | 0.09 | 0.08 | -0.01 | -0.07 | 0.15 | 0.13 |
| **Pericalcerine** | 0.1 | 0.12 | 0.16 | -0.02 | 0.04 | -0.11 | 0.07 | -0.2 |
| **Postcentral** | -0.6 | -0.1 | -0.09 | -0.2 | -0.05 | -0.3 | -0.03 | -0.14 |
| **Posteriorcingulate** | 0.05 | -0.27* | -0.1 | 0.02 | 0.06 | 0.01 | -0.18 | -0.02 |
| **Precentral** | 0.11 | -0.42** | -0.1 | -0.28* | 0.21 | -0.3* | -0.03 | -0.08 |
| **Precuneus** | -0.07 | 0.01 | -0.14 | -0.13 | -0.15 | -0.2 | -0.18 | -0.1 |
| **Rostralanteriorcingulate** | 0.08 | 0.12 | -0.16 | 0.00 | -0.02 | -0.12 | -0.12 | -0.03 |
| **Rostralmiddlefrontal** | 0.14 | 0.17 | 0.16 | 0.04 | 0.11 | 0.26* | 0.08 | 0.07 |
| **Superiorfrontal** | -0.12 | -0.23 | 0.01 | -0.1 | 0.06 | 0.17 | 0.02 | 0.14 |
| **Superiorparietal** | 0.14 | -0.14 | -0.04 | -0.04 | 0.16 | -0.18 | -0.02 | -0.05 |
| **Superiortemporal** | -0.06 | 0.18 | 0.03 | 0.02 | 0.13 | 0.07 | 0.02 | 0.14 |
| **Supramarginal** | -0.09 | -0.25* | -0.05 | -0.2 | -0.06 | -0.18 | 0.09 | -0.13 |
| **Temporalpole** | 0.03 | 0.09 | 0.12 | -0.01 | -0.07 | 0.17 | -0.06 | -0.03 |
| **Transversetemporal** | -0.16 | 0.00 | -0.02 | -0.06 | 0.09 | 0.08 | 0.23 | -0.04 |
| **Cuneus** | 0.06 | 0.15 | -0.07 | 0.2 | 0.01 | -0.04 | -0.07 | 0.24 |
| **Isthmuscingulate** | -0.02 | -0.08 | 0.12 | -0.09 | 0.00 | 0.09 | 0.04 | -0.00 |
| **banksts** | 0.02 | 0.19 | 0.02 | 0.07 | -0.03 | 0.02 | -0.06 | 0.01 |
| **Amygdala** | -0.03 | 0.06 | -0.09 | -0.25* | 0.16 | 0.05 | 0.13 | -0.37** |
| **Hippocampus** | -0.14 | 0.29* | -0.19 | 0.12 | -0.03 | 0.02 | -0.02 | -0.02 |
| **Palladium** | 0.11 | 0.07 | 0.13 | -0.19 | -0.14 | -0.01 | 0.01 | -0.15 |
| **Caudate** | 0.32** | 0.26* | 0.34** | 0.17 | 0.28* | 0.14 | 0.14 | -0.04 |
| **Thalamus** | 0.07 | 0.11 | 0.04 | 0.08 | -0.14 | -0.22 | -0.36** | -0.22 |
| **Brainstem** | -0.21 | -0.09 | -0.21 | -0.09 | -0.14 | 0.01 | -0.14 | 0.01 |
| **Putamen** | 0.19 | -0.08 | 0.35** | -0.01 | 0.22 | -0.07 | 0.08 | -0.1 |
| **Cerebellum** | -0.09 | 0.16 | -0.16 | 0.1 | -0.04 | 0.1 | -0.12 | 0.03 |

Table S7: Pearson correlations between cortical thickness/subcortical volume and Reho and fALFF values in the respective brain region for the left hemisphere. Averaged absolute correlation coefficients did not differ between recAN and HC (fALFF: F(1,82)=1.95,p>0.05; ReHo: F(1,82)=1.12,p>0.05). *=p<0.05, **=p<0.01. fALFF=fractional amplitude of low frequency fluctuations, HC=healthy control, recAN=recovered anorexia nervosa, ReHo=regional homogeneity.

**Table S8: Group comparisons of Cortical Thickness and Subcortical Volume**

|  | **Left Hemisphere** | | | |  | **Right Hemisphere** | | | |
| --- | --- | --- | --- | --- | --- | --- | --- | --- | --- |
| **ROI** | **Mean recAN** | **SD** | **Mean**  **HC** | **SD** | **Mean recAN** | | **SD** | **Mean HC** | **SD** |
| **bankssts** | 2.62 | 0.17 | 2.62 | 2.62 | 2.73 | | 0.16 | 2.76 | 0.19 |
| **caudalanteriorcingulate** | 3.00 | 0.24 | 2.92 | 0.24 | 2.80 | | 0.20 | 2.76 | 0.22 |
| **caudalmiddlefrontal** | 2.71 | 0.16 | 2.70 | 0.16 | 2.68 | | 0.13 | 2.66 | 0.13 |
| **cuneus** | 1.81 | 0.13 | 1.83 | 0.13 | 1.86 | | 0.17 | 1.88 | 0.13 |
| **entorhinal** | 3.44 | 0.35 | 3.41 | 0.40 | 3.70 | | 0.38 | 3.58 | 0.43 |
| **fusiform** | 2.87 | 0.16 | 2.85 | 0.15 | 2.88 | | 0.17 | 2.88 | 0.12 |
| **inferiorparietal** | 2.58 | 0.13 | 2.59 | 0.15 | 2.67 | | 0.12 | 2.65 | 0.13 |
| **inferiortemporal** | 2.97 | 0.18 | 2.93 | 0.16 | 2.95 | | 0.15 | 2.94 | 0.13 |
| **isthmuscingulate** | 2.75 | 0.23 | 2.75 | 0.22 | 2.67 | | 0.20 | 2.68 | 0.23 |
| **lateraloccipital** | 2.27 | 0.15 | 2.27 | 0.13 | 2.36 | | 0.16 | 2.35 | 0.14 |
| **lateralorbitofrontal** | 2.81 | 0.17 | 2.76 | 0.16 | 2.84 | | 0.19 | 2.82 | 0.16 |
| **lingual** | 2.02 | 0.13 | 2.05 | 0.13 | 2.10 | | 0.15 | 2.11 | 0.13 |
| **medialorbitofrontal** | 2.61 | 0.18 | 2.60 | 0.18 | 2.61 | | 0.19 | 2.58 | 0.17 |
| **middletemporal** | 3.09 | 0.17 | 3.05 | 0.18 | 3.14 | | 0.13 | 3.10 | 0.16 |
| **parahippocampal** | 2.97 | 0.34 | 3.04 | 0.38 | 2.97 | | 0.29 | 3.05 | 0.28 |
| **paracentral** | 2.44 | 0.18 | 2.46 | 0.18 | 2.51 | | 0.18 | 2.50 | 0.16 |
| **parsopercularis** | 2.74 | 0.13 | 2.74 | 0.19 | 2.75 | | 0.17 | 2.75 | 0.16 |
| **parsorbitalis** | 2.99 | 0.25 | 2.96 | 0.26 | 2.96 | | 0.25 | 2.97 | 0.23 |
| **parstriangularis** | 2.64 | 0.14 | 2.64 | 0.18 | 2.63 | | 0.17 | 2.63 | 0.17 |
| **pericalcarine** | 1.53 | 0.13 | 1.51 | 0.12 | 1.52 | | 0.12 | 1.54 | 0.12 |
| **postcentral** | 2.14 | 0.13 | 2.14 | 0.11 | 2.09 | | 0.15 | 2.08 | 0.12 |
| **posteriorcingulate** | 2.82 | 0.17 | 2.78 | 0.18 | 2.76 | | 0.17 | 2.70 | 0.16 |
| **precentral** | 2.64 | 0.14 | 2.64 | 0.13 | 2.60 | | 0.16 | 2.58 | 0.15 |
| **precuneus** | 2.49 | 0.16 | 2.49 | 0.18 | 2.50 | | 0.15 | 2.50 | 0.17 |
| **rostralanteriorcingulate** | 3.25 | 0.22 | 3.19 | 0.24 | 3.09 | | 0.23 | 3.06 | 0.23 |
| **rostralmiddlefrontal** | 2.57 | 0.14 | 2.55 | 0.15 | 2.49 | | 0.12 | 2.47 | 0.14 |
| **superiorfrontal** | 2.99 | 0.14 | 2.96 | 0.16 | 2.94 | | 0.14 | 2.93 | 0.15 |
| **superiorparietal** | 2.22 | 0.13 | 2.24 | 0.15 | 2.24 | | 0.13 | 2.24 | 0.14 |
| **superiortemporal** | 2.96 | 0.19 | 2.98 | 0.17 | 3.02 | | 0.16 | 3.02 | 0.15 |
| **supramarginal** | 2.69 | 0.16 | 2.70 | 0.15 | 2.73 | | 0.14 | 2.70 | 0.14 |
| **frontalpole** | 3.08 | 0.34 | 3.08 | 0.36 | 3.05 | | 0.35 | 3.03 | 0.36 |
| **temporalpole** | 3.81 | 0.38 | 3.77 | 0.35 | 3.97 | | 0.32 | 3.92 | 0.44 |
| **transversetemporal** | 2.54 | 0.27 | 2.49 | 0.25 | 2.55 | | 0.23 | 2.53 | 0.27 |
| **insula** | 3.21 | 0.14 | 3.27 | 0.15 | 3.22 | | 0.15 | 3.24 | 0.16 |
| **cerebellum** | 58.36 | 49.37 | 58.27 | 41.63 | 60.66 | | 44.80 | 60.02 | 43.17 |
| **thalamus** | 8.66 | 8.35 | 8.92 | 8.33 | 7.92 | | 6.95 | 8.11 | 6.66 |
| **caudate** | 4.00 | 5.04 | 4.01 | 5.11 | 3.86 | | 4.19 | 3.86 | 4.59 |
| **putamen** | 5.48 | 6.09 | 5.68 | 5.78 | 5.23 | | 5.66 | 5.34 | 5.23 |
| **pallidum** | 1.47 | 2.22 | 1.54 | 2.26 | 1.43 | | 1.57 | 1.49 | 1.66 |
| **hippocampus** | 4.27 | 3.69 | 4.32 | 3.21 | 4.28 | | 4.03 | 4.38 | 3.19 |
| **amygdala** | 1.54 | 1.80 | 1.58 | 1.72 | 1.51 | | 1.83 | 1.56 | 1.53 |

Table S8: Mean and standard deviations of cortical thickness and subcortical volume data using the ROIs of the Desikan Killiany Atlas for each hemisphere. Volume measurements are displayed as raw values (mm3). After Bonferroni correction for multiple testing, none of the comparisons using an independent t-test were significant (uncorrected p=0.015-0.996). RecAN=recovered anorexia nervosa, HC=healthy control, SD=standard deviation.
